# Acute Innominate Vein Dissection During Pacemaker Revision Procedure: A Case Report and Conservative Management

**DOI:** 10.7759/cureus.107901

**Published:** 2026-04-28

**Authors:** Frank Jorge Valdez Baez, Cemirame Payan Jimenez, Catherine Merejo, Juanico Cedano Ramirez, Luisa Paulino Lopez, Warenny Montero Morillo, Evelina Severino Marte

**Affiliations:** 1 Electrophysiology, Asociacion Instituto Dominicano de Cardiologia, Santo Domingo, DOM; 2 Imaging and Computed Tomography, Asociacion Instituto Dominicano de Cardiologia, Santo Domingo, DOM; 3 Anesthesiology, Asociacion Instituto Dominicano de Cardiologia, Santo Domingo, DOM

**Keywords:** iatrogenic complication, innominate vein, pacemaker, venous dissection, venous thrombosis

## Abstract

Iatrogenic central venous dissection during cardiac pacing procedures is exceptional. We present the case of a 75-year-old woman with a single-chamber pacemaker since 2004, who underwent generator replacement and new ventricular lead implantation in 2025. During the procedure, anomalous resistance to lead advancement was identified. Immediate venography revealed innominate vein dissection with intramural hematoma. The procedure was discontinued, and the patient remained stable. Computed tomography angiography at 24 hours confirmed complete occlusion of the left subclavian and innominate veins due to thrombotic progression. Conservative management with deferred anticoagulation was adopted after confirming clinical stability and absence of pericardial effusion progression. Given that the patient was not pacemaker-dependent (ventricular pacing, 2.6%) and the existing ventricular lead retained adequate electrical parameters, a new generator was connected to the previous lead without adding a new lead, deferring any further transvenous implantation attempt to avoid additional manipulation of the recently injured venous system. The patient evolved favorably, with confirmation of right contralateral access patency as a viable alternative for potential future implantation if clinically required. This case illustrates the importance of early recognition of acute venous injuries and individualization of therapeutic management.

## Introduction

Transvenous implantation of permanent pacemakers is the standard treatment for bradyarrhythmias, preferably performed via subclavian access. Although it is a safe procedure, intravascular leads can induce alterations in the central veins, resulting in thrombosis, stenosis, or occlusion of the subclavian vein, brachiocephalic (innominate) vein, or superior vena cava (SVC) [1]. These complications typically develop progressively over months or years. Early after lead insertion, endothelial damage and perturbation of blood flow trigger a prothrombotic reaction mediated by neutrophils, macrophages, and foreign body giant cells, with elevation of circulating procoagulant factors including von Willebrand factor, prothrombin fragment, plasminogen activator inhibitor-1, and D-dimer. Long-term lead residence leads to progressive vein wall thickening, fibrous incorporation of the lead into the intima, and perivascular fibrous hyperplasia [2]. Venographic series report that 30-45% of pacemaker patients present some degree of central venous obstruction, although only 0.6% to 3.5% of individuals develop serious thrombotic and embolic complications [3,4]. When obstruction is significant, severe complications such as SVC syndrome may occur [3].

Acute venous occlusion during the implantation procedure is extremely rare. Described predisposing factors include the presence of multiple leads, history of venous thrombosis, previous systemic infections, and anatomical variations. Left-sided access, with its more angulated trajectory toward the SVC, carries a greater risk of vascular trauma [5,6]. Recent evidence further suggests that periprocedural complication profiles in cardiac implantable electronic devices may differ by sex: in a contemporary propensity-score-matched cohort of more than 20,000 patients, men showed a higher incidence of vascular complications, while women showed a higher risk of cardiac and pulmonary complications [7]. This observation, relevant for preprocedural planning and individualized risk assessment, provides additional context for the case we describe.

Beyond the chronic processes described above, a distinct clinical scenario is that of acute mechanical injury to the venous wall occurring during the access phase itself, when the guidewire or subsequent instruments enter a submural plane from the initial puncture and propagate a dissection tract within the venous wall rather than advancing through the true lumen. Unlike chronic lead-related venous obstruction, this mechanism involves a primary structural injury to the vein occurring within seconds to minutes, with secondary thrombotic progression over the ensuing hours rather than months. Such events are rarely reported in the literature, likely because they are either immediately recognized and aborted without significant sequelae or because the dissection planes evolve silently and are interpreted retrospectively as thrombosis of unclear origin.

Early diagnosis requires a high index of suspicion and appropriate use of imaging. Venography remains the reference standard, while computed tomography angiography defines the extent of damage and presence of collateral circulation [8]. We present a case in which an acute iatrogenic dissection, most likely initiated by submural guidewire positioning during puncture and subsequently amplified by dilator passage and pressurized contrast injection, progressed within 24 hours to complete thrombotic occlusion of the left subclavian and innominate veins, highlighting the specific pathophysiology of acute access-related venous injury and the individualization of its therapeutic management.

## Case presentation

Relevant background

The diagnostic clues central to this case, unexpected mechanical resistance to lead advancement despite prior uncomplicated guidewire passage, fluoroscopic trajectory deviation not visible until contrast confirmation, and rapid thrombotic progression to complete venous occlusion within 24 hours, emerged sequentially during a revision procedure in a patient with a long-standing pacemaker system, as described below.

A 75-year-old hypertensive woman with a documented history of lower-limb peripheral arterial disease, carrier of a VVI single-chamber pacemaker since August 2004 (Medtronic Sigma; Medtronic, Inc., Mounds View, MN, USA) with generator replacement in 2017 (Medtronic Sensia VVI; Medtronic, Inc., Mounds View, MN, USA), preserving the original ventricular lead. The original indication for pacemaker implantation in 2004 could not be documented, as the procedure was performed at a different institution and the initial clinical records were not available for review. However, the consistently low ventricular pacing burden observed throughout long-term follow-up (2.6% at the time of device evaluation in 2025), together with preserved spontaneous atrioventricular conduction over 21 years, is consistent with sinus node dysfunction as the most probable underlying indication. Serial echocardiographic follow-up over a 10-year period consistently demonstrated preserved left ventricular ejection fraction (LVEF) with left ventricular hypertrophy. Measurements ranged predominantly between 64% and 76%, with the most recent assessment performed by the 2D biplane Simpson method yielding 69%, and a prior invasive ventriculography in 2016 documenting an LVEF of 65% together with angiographically normal epicardial coronary arteries without evidence of obstructive disease. An isolated higher value recorded in one intermediate study likely reflected methodological variation, as the imaging technique was not consistently documented across studies. The patient remained asymptomatic under chronic treatment for over 10 years with losartan 100 mg once daily, bisoprolol 2.5 mg once daily, and clopidogrel 75 mg once daily, the latter prescribed as long-term antiplatelet therapy for her documented peripheral arterial disease.

Procedure indication

In August 2025, device interrogation documented a battery voltage of 2.72 V with an estimated longevity of three months and a ventricular pacing percentage of 2.6%. Measured electrical parameters of the ventricular lead were within normal operating ranges (sensed R-wave amplitude 8 mV, pacing threshold 0.7 V, lead impedance 601 Ω). Notwithstanding these normal punctual values, a 24-hour Holter recording evidenced intermittent ventricular undersensing with functional loss of capture and fusion beats, indicating clinically relevant sensing dysfunction during ambulatory monitoring. Considering the documented sensing dysfunction on Holter, the pending battery depletion, and the 21-year permanence of the pacing system, generator replacement and new ventricular lead implantation were decided.

Procedure description

The procedure was performed as an elective intervention under sterile conditions and local anesthesia. In preparation for the procedure, chronic antiplatelet therapy with clopidogrel had been discontinued five days in advance, following standard perioperative recommendations for elective device interventions in patients under chronic antiplatelet therapy for peripheral vascular disease. An incision was made in the left infraclavicular region over the previous pacemaker pocket, followed by dissection through tissue planes to expose the generator, which was removed and disconnected. The ventricular lead was freed, documenting adequate electrical parameters with an R-wave amplitude of 9.8 mV and impedance of 466 ohms.

Subsequently, venous access was obtained via the left infraclavicular route using the Seldinger technique, guided by the preceding diagnostic venography, which showed no signs of venous occlusion or stenosis. Venous puncture was performed using standard anatomical landmarks and the venogram as a reference, in single-plane anteroposterior fluoroscopic projection. Successful venous cannulation was confirmed by the expected non-pulsatile dark blood return through the needle. A standard 0.035-inch J-tipped spring guidewire was then advanced without resistance, with the tip reaching the right atrial silhouette along a fluoroscopically adequate trajectory. No additional methods of guidewire position confirmation were employed, as there were no intraprocedural signs suggesting an abnormal course at that time.

Next, a 7-French peel-away introducer sheath with its corresponding dilator was introduced. After removing the dilator, an attempt was made to advance a new ventricular lead (Medtronic model 4076, 58 cm, bipolar, active-fixation; Medtronic, Inc., Mounds View, MN, USA) through the introducer sheath, with unexpected mechanical opposition to its progression observed, associated with an anomalous trajectory under fluoroscopy. Given this finding, the lead was withdrawn, and a targeted contrast injection was performed in anteroposterior projection through the introducer sheath, consisting of a single bolus of approximately 10 mL administered by manual push. The venographic acquisition revealed anomalous opacification that did not follow the usual trajectory toward the innominate vein and SVC. A false medial tract was identified, compatible with venous dissection and contrast accumulation in an intramural hematoma, with contrast trajectory suggestive of possible extension toward the right atrial wall region (Video 1).

**Video 1:** Fluoroscopic sequence showing contrast extravasation and left innominate vein dissection during lead advancement Fluoroscopic sequence in anteroposterior projection evidencing progressive contrast extravasation toward the superior mediastinum following left subclavian vein opacification. A contrast column is observed dissecting the venous wall and delineating an intramural hematoma, indicative of iatrogenic dissection of the left brachiocephalic venous trunk. Persistence of the filling defect and anomalous deviation of contrast flow support the presence of a significant structural lesion in the venous pathway

In this context, and to avoid progression of the venous injury, no additional attempts at intravascular material advancement were made. The introducer sheath was removed, and a new generator (Medtronic Astra S, SR MRI SureScan; Medtronic, Inc., Mounds View, MN, USA) was connected to the previous ventricular lead, which was programmed in VVI mode at 40 beats per minute. The generator was placed in a new surgical pocket, and the wound was closed in layers without additional incidents.

Immediate evolution

**Figure 1:**
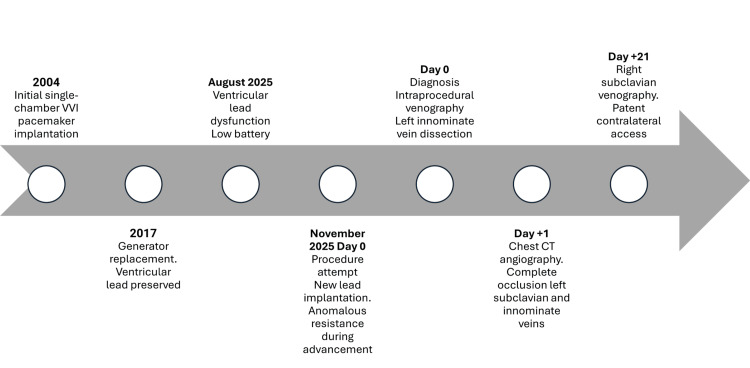
Clinical timeline of relevant events Chronological timeline of the clinical course, spanning 21 years from initial pacemaker implantation in 2004 to the three-month follow-up after the iatrogenic venous complication. The timeline is designed to allow rapid orientation to the temporal sequence of events and their relative intervals, emphasizing the long-standing nature of the pacing system and the acute onset of the intraprocedural complication

The clinical evolution and temporal sequence of post-procedure events are summarized in Figure 1. The patient remained asymptomatic and hemodynamically stable throughout the post-procedure observation period, with specific absence of the clinical manifestations that would typically be expected from acute subclavian and innominate vein occlusion: no left upper extremity swelling or edema, no perceptible asymmetry in limb circumference compared to the contralateral side, no cyanosis or abnormal coloration, no chest discomfort or dyspnea, no visible superficial collateral circulation, and no jugular venous engorgement. Blood pressure remained symmetric in both upper extremities. Post-procedure echocardiogram showed mild pericardial effusion without signs of tamponade, interpreted as a reactive response to trauma. Given the recent vascular injury and effusion, immediate parenteral anticoagulation was avoided, adopting close clinical observation.

**Figure 2:**
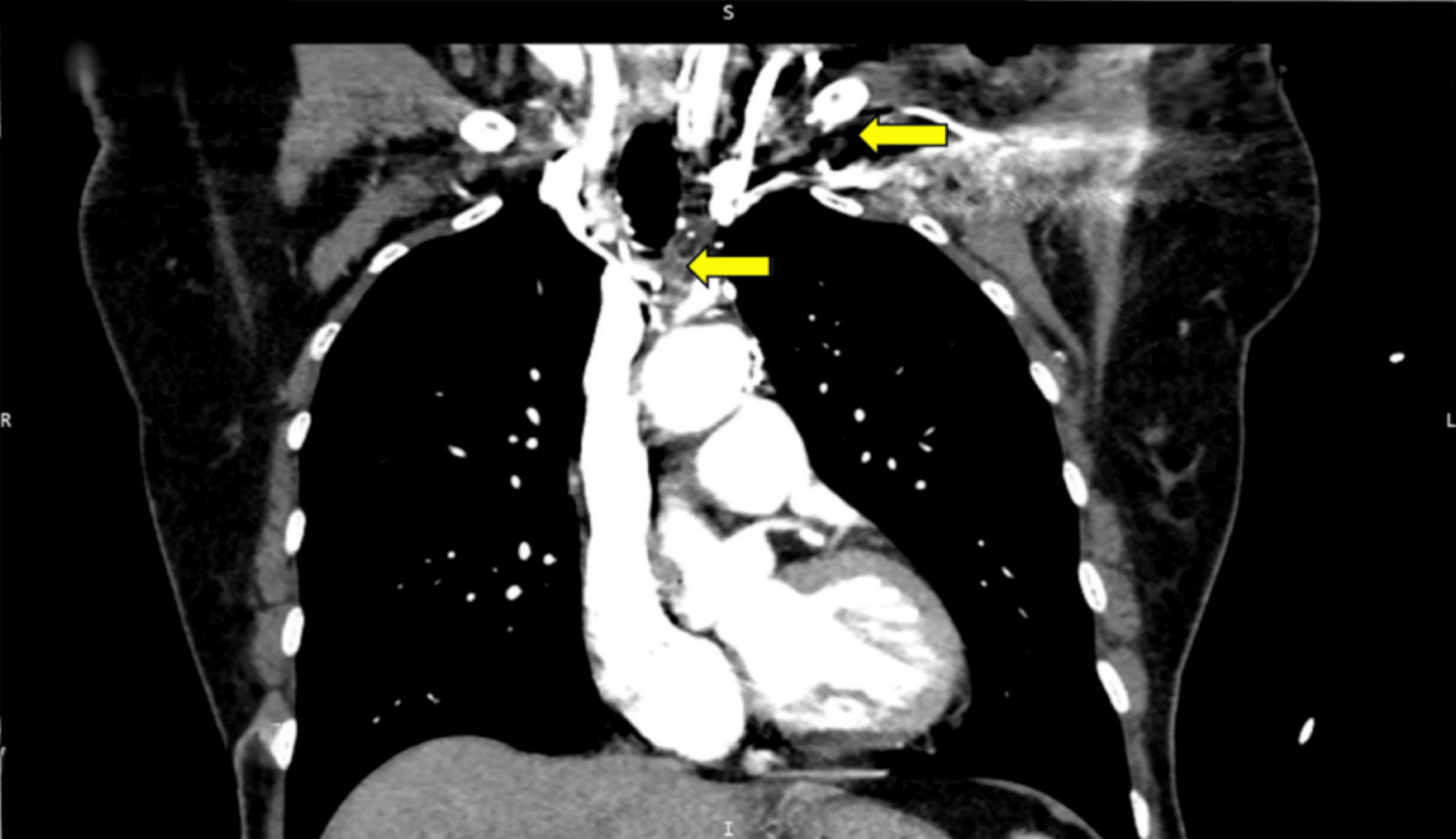
Coronal reconstruction of chest computed tomography angiography showing complete occlusion of the left subclavian and innominate veins Coronal reconstruction of chest computed tomography angiography in venous phase. The upper arrow indicates the expected anatomical location of the left subclavian vein, demonstrating complete absence of contrast opacification. The lower arrow indicates the expected anatomical location of the left brachiocephalic (innominate) venous trunk, similarly showing absence of opacification. These findings are compatible with complete venous occlusion secondary to iatrogenic dissection. A marked asymmetry is observed when compared with the normal opacification of the contralateral right-sided venous system, which serves as an internal anatomical reference. The superior vena cava remains patent, ensuring preserved central venous return through the right-sided pathway. Systematic pericardial assessment on this study documented the absence of significant pericardial fluid collection, with normal visualization of the pericardial layers and no focal pericardial thickening, loculated effusions, or localized hematoma, confirming the resolution of the mild pericardial effusion detected echocardiographically in the immediate post-procedure period

Computed tomography angiography the following day (Figure 2) revealed complete occlusion of the left subclavian and innominate veins, compatible with thrombotic progression of the intramural dissection. The SVC remained patent. No mediastinal lymphadenopathy, pulmonary infiltrates, pleural effusions, or pneumothorax was identified. The pericardial effusion was not visualized, suggesting resolution of the echocardiographic finding.

The patient remained under observation for four days without complications. Serial hematological monitoring documented a clinically significant decrease in hemoglobin from a pre-procedure baseline of 12.9 g/dL (obtained several months earlier) to a post-event nadir of 10.1 g/dL on the second post-procedure day, representing an approximate 2.8 g/dL drop compatible with contained acute bleeding into the intramural hematoma. Hemoglobin subsequently stabilized spontaneously without the need for transfusion, with values of 10.6, 10.1, 10.7, and 10.5 g/dL on days 1, 2, 3, and 4 post-procedure, respectively. Hematocrit remained between 32.0% and 32.1%, and platelet count was between 238 and 255 ×10³/µL throughout the observation period. The pattern of rapid initial decline followed by spontaneous stabilization without ongoing hemoglobin drop was consistent with self-limited bleeding into the established intramural hematoma, without evidence of continued active hemorrhage.

Therapeutic management and follow-up

After confirming clinical and echocardiographic stability, ruling out pericardial effusion progression, the patient was discharged with deferred oral anticoagulation: apixaban 10 mg every 12 hours for seven days, then 5 mg every 12 hours, according to standard dosing for acute venous thrombosis. A total anticoagulation course of three months was planned, in accordance with current guideline recommendations for provoked venous thrombosis associated with a transient major factor; in this case, the iatrogenic vascular injury, with the expectation that the provoking factor would resolve as the venous wall healed and the intramural hematoma reorganized, and without a persistent prothrombotic condition justifying indefinite therapy.

The decision to defer anticoagulation until discharge was based on the following: (1) presence of pericardial effusion in the first post-procedure hours with risk of hemorrhagic expansion, (2) recent acute venous dissection with potential for intramural bleeding, (3) hemodynamic stability without evidence of pulmonary embolism, and (4) need for observation to rule out vascular lesion progression. Once clinical stability and pericardial effusion resolution were confirmed, it was considered that the benefit of anticoagulation to prevent thrombotic extension outweighed the residual hemorrhagic risk. Clopidogrel, which had been discontinued five days before the procedure, was not resumed at any point following the complication, given the initial bleeding-related contraindications and, subsequently, the unfavorable risk-benefit balance of adding antiplatelet therapy to ongoing anticoagulation with apixaban in a patient with a recent venous dissection.

**Figure 3:**
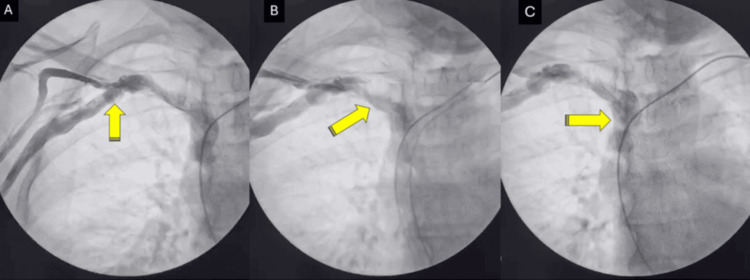
Fluoroscopic sequence of venous access via right subclavian route Temporal sequence in anteroposterior projection documenting intact patency of the contralateral venous system. (A) Initial phase showing opacification of the proximal right subclavian vein (arrow). (B) Contrast progression toward the right innominate vein (arrow). (C) Late phase evidencing continuous flow toward the superior vena cava (arrow). Uniform contrast passage without filling defects, stenosis, or obstruction confirms the anatomical viability of right-sided access for future procedures. The previous pacemaker lead is visible as a radiopaque structure

At 15 days, the patient returned asymptomatic with adequate surgical healing and no complications. At 21 days, diagnostic venography was performed via the right subclavian route (Figure 3), demonstrating intact patency of the contralateral venous pathway, confirming anatomical viability for a potential future procedure. The pacemaker was reprogrammed to VVI mode, correcting the sensing alteration.

Given that the patient was not pacemaker-dependent (ventricular pacing 2.6%) and considering the venous dissection in the subacute phase with thrombotic progression, any additional transvenous implantation attempt was deferred. This strategy sought to (1) avoid manipulation of the recently injured venous system, (2) minimize the risk of dissection extension, (3) allow anatomical evaluation of contralateral access, and (4) maintain a close follow-up to plan deferred implantation if necessary.

At three months, control studies were performed to evaluate evolution and rule out embolic complications, with the patient still on ongoing apixaban therapy at the time of imaging. Venous Doppler of the upper extremities showed persistence of altered venous flow in the brachial and ulnar segments of the left upper extremity, without evidence of active thrombosis or residual thrombotic material, with mild bilateral distal soft tissue edema with left predominance compatible with post-thrombotic sequelae. Chest CT angiography ruled out pulmonary thromboembolism, with a pacemaker lead in adequate position in the right cardiac chambers without data of perforation or rupture, atheromatous thoracic aorta with nonsignificant calcified plaques, and bilateral basal fibroatelectatic pleuro-pulmonary bands without pleural effusion. Based on these favorable imaging findings and the completion of the planned three-month therapeutic course, apixaban was discontinued at that point, and clopidogrel 75 mg once daily was resumed for continued antiplatelet protection of the underlying peripheral arterial disease, returning the patient to her original long-term antithrombotic regimen.

## Discussion

This case documents an exceptional complication: acute complete occlusion of the subclavian and innominate veins secondary to iatrogenic dissection during pacemaker manipulation. High-grade obstruction involving one or two veins simultaneously has been reported in 38.4% and 22.2% of device carriers, respectively, while severe SVC involvement remains rare, occurring in less than 0.9% of cases [6]. Symptomatic obstruction is far less common, accounting for less than 0.5% of patients with transvenous pacemaker leads, and typically develops over months to years rather than acutely [9]. The immediate intraprocedural occurrence in this patient underscores the singular nature of this event.

Mechanism of injury

Acute venous injuries during lead implantation can occur through various mechanisms. In this case, dissection with intramural hemorrhage resulted from direct mechanical trauma to the vascular wall during venous access instrumentation.

Retrospective analysis of the sequence of events suggests a cumulative mechanism that may have originated from the initial phase of the procedure. Although venous puncture was apparently successful and the guidewire advanced without resistance, the absence of resistance during guidewire passage does not exclude extraluminal positioning: a submural dissection plane may develop easily within the media of the venous wall, approximately following the anatomical course of the vessel until reaching the right atrial silhouette, with a fluoroscopic trajectory misleadingly consistent with intraluminal position. Fluoroscopic trajectory alone cannot reliably distinguish an intraluminal from a submural course once the guidewire tip reaches the cardiac silhouette.

Under this interpretation, the pathophysiological sequence would have involved the following: (1) initial positioning of the guidewire within a submural plane that was not detected fluoroscopically; (2) enlargement of the dissecting tract during introduction of the 7-French dilator and its sheath, which, as rigid instruments of greater caliber, would have deepened and extended the dissection; and (3) clinical manifestation upon attempted advancement of the ventricular lead, whose greater longitudinal stiffness and effective profile encountered mechanical opposition while attempting to progress through the established false tract. Confirmatory contrast injection, delivered as a single approximately 10 mL push through the introducer sheath positioned within the dissection plane, then delineated the intramural hematoma and the anomalous course. Pressurized administration of contrast within a dissection space may have acted as a hydraulic dissecting agent, mechanically propagating the dissection plane and increasing the volume of the intramural hematoma. Although its specific contribution cannot be retrospectively quantified, we consider it a probable amplifying factor within the pathophysiological sequence.

The absence of obstructions on pre-procedure venography indicates that the injury was not a consequence of a previously compromised venous pathway. Progression to complete thrombotic occlusion within the first 24 hours represents an immediate sequela of endothelial damage with activation of the coagulation cascade and thrombus formation over the intramural hematoma, a mechanism well-described in the early response of the vein wall to indwelling devices [2]. Notably, large-scale evidence suggests that the mechanical impact of lead burden on the vein wall plays a central role in determining the severity of venous obstruction, potentially outweighing purely thrombotic mechanisms, particularly long after implantation [10].

Predisposing factors in this case

Several factors may have contributed to this complication: (1) left-sided access, whose more angulated trajectory toward the SVC increases the risk of mechanical trauma during instrumentation [6]; (2) presence of a ventricular lead with 21 years of intravascular permanence, sufficient time to generate significant perivascular fibrosis that reduces venous distensibility and creates adhesions that limit the effective space available for new material passage [3]; and (3) the need to simultaneously accommodate two leads through the same venous pathway, a factor consistently associated with higher risk of venous obstruction. Multiple studies have demonstrated a significant association between lead number, sum of lead diameters, and venous stenosis [11-13], and multivariate analysis in a large series of 2,909 patients confirmed that the number of leads (OR: 1.35; p = 0.003) and the presence of abandoned leads (OR: 1.97; p < 0.001) were independent predictors of lead-related venous stenosis/obstruction (LRVSO) severity [10], and (4) although pre-procedure venography showed no obstructions, studies demonstrate that 13.7% of patients present asymptomatic venous stenoses before new implants, especially at the innominate-SVC junction [5]. Additional predisposing factors described in the literature include prior intravascular lead infection and the use of a temporary transvenous wire before implantation, both associated with increased risk of venous stenosis and obstruction [14,15].

Critical diagnostic aspects

Complication detection relied on the integration of intraprocedural clinical and imaging findings. Unexpected mechanical resistance to lead advancement, absent during prior guidewire and dilator passage, constituted the first warning sign, prompting immediate fluoroscopic assessment and confirmatory venography. This decision was crucial in limiting the extent of injury, as additional manipulation attempts could have resulted in complete vascular wall perforation, massive extravasation, or dissection extension toward mediastinal or atrial structures.

Computed tomography angiography at 24 hours served dual diagnostic and decision-making purposes: it confirmed complete subclavian and innominate occlusion, excluded hemothorax and mediastinal collections, documented SVC patency, and demonstrated pericardial effusion resolution. Each finding directly guided management, patent SVC supported conservative over endovascular intervention, and effusion resolution enabled safe anticoagulation initiation at discharge.

Consideration of atrial extension

Possible extension toward the right atrial wall was raised as an intraprocedural concern based exclusively on the fluoroscopic trajectory of the extravasated contrast, which appeared to approach the right atrial silhouette in anteroposterior projection. This observation should be interpreted with caution, as fluoroscopic two-dimensional imaging cannot reliably distinguish true atrial wall involvement from contrast tracking along the venous dissection plane approaching the atrial silhouette through superimposed anatomical structures. No definitive imaging evidence of atrial wall injury was obtained: computed tomography angiography at 24 hours did not demonstrate structural atrial wall disruption, pericardial extravasation, or localized pericardial hematoma, and cardiac magnetic resonance imaging, which would provide greater sensitivity for atrial wall assessment, was not pursued given the favorable clinical and echocardiographic evolution. The mild pericardial effusion detected echocardiographically in the immediate post-procedure period may have resulted from either limited extravasation from the venous dissection into the pericardial space or inflammatory pericardial reaction to mediastinal trauma; its spontaneous resolution within 24 hours, together with hemodynamic stability throughout the clinical course, argues strongly against significant structural atrial involvement. The concern for atrial extension, therefore, remained a theoretical consideration derived from intraprocedural fluoroscopic appearance rather than a confirmed imaging finding. This scenario underscores the importance of serial echocardiographic monitoring in the post-procedure period, while acknowledging that the absence of definitive tomographic evidence of atrial injury argues against clinically significant atrial wall involvement in this case.

Justification for conservative management

Given the low frequency of these complications, no consensus exists regarding optimal treatment, and available evidence is largely limited to case series and anecdotal experience; randomized trials addressing the management of lead-related venous occlusion remain lacking [16,17]. When lead-related venous obstruction becomes symptomatic, anticoagulation represents the established first-line medical approach: initiated early, it can relieve acute venous obstruction and prevent thrombus propagation toward the innominate vein and SVC, reducing the risk of SVC syndrome and pulmonary embolization [3]. When medical therapy alone proves insufficient, interventional strategies, such as venoplasty combined with lead extraction, thoracoscopic surgery, or surgical reconstruction, have demonstrated efficacy in symptomatic or hemodynamically compromised patients [1,18-21].

The clinical course of this case, however, supported a more conservative individualized approach. The initial presentation was dominated by an intramural hematoma with associated pericardial effusion, both of which constituted active contraindications to immediate anticoagulation. Crucially, the patient remained asymptomatic throughout despite angiographically documented complete occlusion.

This clinico-angiographic dissociation is itself informative at two levels. Pathophysiologically, the absence of venous hypertension symptoms indicates that effective hemodynamic compensation was already present at the time of imaging, either through early collateral recruitment or through residual venous drainage not fully captured by cross-sectional imaging, a recognized limitation of CT angiography in the evaluation of dynamic venous flow [3]. Clinically, this dissociation also informs therapeutic decision-making: in the absence of clinical venous hypertension, the expected benefit of aggressive interventional strategies, such as endovascular recanalization or surgical reconstruction, would be modest, while their procedural risks remain meaningful, tilting the risk-benefit balance toward conservative management in this scenario. Anticoagulation was, therefore, deferred until thrombotic progression became the predominant finding and hemodynamic stability was confirmed, at which point apixaban was initiated at standard dosing for acute venous thrombosis. This individualized strategy is consistent with reported experience showing that anticoagulation with direct oral anticoagulants can achieve complete resolution of pacemaker-induced venous obstruction in patients managed without invasive procedures, including cases complicated by intramural hematoma [16,17]. Contralateral implantation remains the safest option to restore dual-chamber pacing if necessary, and venographic confirmation of right-sided access patency validates this strategy.

Context in the literature

The literature describes very few cases with similar characteristics. Most correspond to SVC or right innominate confluence injuries, presenting subacutely as SVC syndrome. Isolated reports of left innominate vein perforation and thrombosis exist, although generally linked to chronic occlusions with established collateral circulation [20,21]. The combination of acute dissection with immediate total occlusion has been described only anecdotally, accentuating the value of this report.

Practical implications

Vascular trauma during pacemaker revision procedures can be cumulative, with successive procedural steps amplifying an initially subclinical injury. Several practical recommendations emerge from this experience. Before revision procedures in carriers of long-standing leads, pre-procedure venography should be routine to identify asymptomatic central stenoses, and lead function should be evaluated by integrating in-office electrical measurements with ambulatory Holter monitoring, as intermittent dysfunction may escape single-point interrogation. Risk stratification should consider systemic vascular phenotype, including peripheral arterial disease, and sex-based differences in complication profiles. Chronic antiplatelet therapy should be interrupted according to standard perioperative recommendations for elective interventions with moderate bleeding risk, with post-procedural resumption individualized against the evolving antithrombotic balance.

Intraprocedurally, fluoroscopic surveillance should extend throughout all phases of instrumentation. Because fluoroscopic trajectory alone cannot distinguish intraluminal from submural courses once the guidewire reaches the cardiac silhouette, intermediate venography after guidewire passage and before dilation should be considered, particularly in revisions where perivascular fibrosis distorts venous anatomy. If dissection is suspected, diagnostic contrast should be administered with minimum volume and slow controlled injection, avoiding pressurized push through introducer sheaths or dilators already positioned in the dissection plane. Once a vascular complication occurs, management should be guided by clinical stability, imaging findings, and pacing dependence; individualized conservative strategies with deferred anticoagulation may be appropriate in stable patients with favorable anatomical evolution, reserving interventional or surgical approaches for refractory cases or hemodynamic compromise [1].

Evolution at three-month follow-up

The three-month follow-up confirmed the expected trajectory of conservative management: thrombus resolution without embolic complications, persistent but stable post-thrombotic sequelae in the left upper extremity, and maintained lead integrity. Imaging studies, performed while the patient was still on apixaban, documented the absence of residual active thrombosis and the absence of pulmonary embolism. Upon completion of the planned three-month therapeutic course, anticoagulation was discontinued, and chronic antiplatelet therapy with clopidogrel was resumed for the underlying peripheral arterial disease. This evolution validates the decision to defer more aggressive interventions given minimal device dependence and clinical stability throughout.

Limitations

This report documents the acute evolution and initial management of the complication with a three-month follow-up. Imaging studies performed at three months post-event confirm thrombus resolution and the absence of embolic complications. However, functional studies of the left upper extremity (plethysmography, provocative venography) that would allow objective quantification of the degree of hemodynamic compensation achieved and long-term venous functionality are not available. Additional imaging follow-up at 6-12 months is scheduled to evaluate the evolution of venous remodeling and complete development of collateral circulation.

Endovascular interventions (directed thrombolysis or stenting) or surgical approaches were not performed due to favorable evolution with conservative management, limiting comparative evaluation of these strategies in this specific context. The absence of significant pacemaker dependence (ventricular pacing 2.6%) facilitated the decision to defer new lead implantation, a strategy that may not be applicable in patients with greater pacing dependence.

## Conclusions

This report contributes to the existing literature on lead-related venous complications in several specific ways. First, it documents a combination of findings not previously described in the indexed literature: acute iatrogenic venous dissection with intramural hematoma, progressing to complete thrombotic occlusion of the subclavian and innominate veins within 24 hours during a revision procedure. Second, it illustrates a striking clinico-angiographic dissociation, persistent asymptomatic status despite angiographically documented complete occlusion, a phenomenon typical of chronic occlusions now documented acutely. Third, it demonstrates that an individualized conservative strategy can be successful in a narrowly defined clinical scenario and delineates the specific circumstances that justify this approach: hemodynamic stability, pericardial effusion resolution, minimal pacing dependence, and confirmed contralateral patency. This recommendation is scenario-specific and not generalizable: in patients with high pacing dependence, such as complete atrioventricular block, the inability to defer reimplantation would shift the risk-benefit balance, and alternative strategies (contralateral implantation without delay, endovascular recanalization, or surgical approaches) would require earlier consideration.

Beyond documenting these findings, the case yields practical recommendations not systematically addressed in previous reports, intermediate confirmatory venography after guidewire passage and specific precautions for contrast administration when dissection is suspected, and integrates contemporary considerations relevant to individualized risk stratification in cardiac device procedures, including systemic vascular phenotype and sex-based differences in complication profiles. The report also contributes to the limited Latin American literature on exceptional electrophysiological complications, broadening the geographical diversity of case reporting in this field.
